# Global analysis of the biosynthetic chemical space of marine prokaryotes

**DOI:** 10.1186/s40168-023-01573-3

**Published:** 2023-06-28

**Authors:** Bin Wei, Gang-Ao Hu, Zhen-Yi Zhou, Wen-Chao Yu, Ao-Qi Du, Cai-Ling Yang, Yan-Lei Yu, Jian-Wei Chen, Hua-Wei Zhang, Qihao Wu, Qi Xuan, Xue-Wei Xu, Hong Wang

**Affiliations:** 1 College of Pharmaceutical Science & Collaborative Innovation Center of Yangtze River Delta Region Green Pharmaceuticals, Key Laboratory of Marine Fishery Resources Exploitment & Utilization of Zhejiang Province, Zhejiang University of Technology, Hangzhou, 310014 China; 2 Key Laboratory of Marine Ecosystem and Biogeochemistry, Ministry of Natural Resources & Second Institute of Oceanography, Ministry of Natural Resources, Hangzhou, 310012 China; 3 Department of Chemistry, Institute of Biomolecular Design & Discovery, Yale University, West Haven, CT 06516 USA; 4 Institute of Cyberspace Security, College of Information Engineering, Zhejiang University of Technology, Hangzhou, 310023 China

**Keywords:** Marine prokaryotes, Biosynthetic gene clusters, Secondary metabolite, Genomics, Cheminformatics

## Abstract

**Background:**

Marine prokaryotes are a rich source of novel bioactive secondary metabolites for drug discovery. Recent genome mining studies have revealed their great potential to bio-synthesize novel secondary metabolites. However, the exact biosynthetic chemical space encoded by the marine prokaryotes has yet to be systematically evaluated.

**Results:**

We first investigated the secondary metabolic potential of marine prokaryotes by analyzing the diversity and novelty of the biosynthetic gene clusters (BGCs) in 7541 prokaryotic genomes from cultivated and single cells, along with 26,363 newly assembled medium-to-high-quality genomes from marine environmental samples. To quantitatively evaluate the unexplored biosynthetic chemical space of marine prokaryotes, the clustering thresholds for constructing the biosynthetic gene cluster and molecular networks were optimized to reach a similar level of the chemical similarity between the gene cluster family (GCF)-encoded metabolites and molecular family (MF) scaffolds using the MIBiG database. The global genome mining analysis demonstrated that the predicted 70,011 BGCs were organized into 24,536 mostly new (99.5%) GCFs, while the reported marine prokaryotic natural products were only classified into 778 MFs at the optimized clustering thresholds. The number of MF scaffolds is only 3.2% of the number of GCF-encoded scaffolds, suggesting that at least 96.8% of the secondary metabolic potential in marine prokaryotes is untapped. The unexplored biosynthetic chemical space of marine prokaryotes was illustrated by the 88 potential novel antimicrobial peptides encoded by ribosomally synthesized and post-translationally modified peptide BGCs. Furthermore, a sea-water-derived *Aquimarina* strain was selected to illustrate the diverse biosynthetic chemical space through untargeted metabolomics and genomics approaches, which identified the potential biosynthetic pathways of a group of novel polyketides and two known compounds (didemnilactone B and macrolactin A 15-ketone).

**Conclusions:**

The present bioinformatics and cheminformatics analyses highlight the promising potential to explore the biosynthetic chemical diversity of marine prokaryotes and provide valuable knowledge for the targeted discovery and biosynthesis of novel marine prokaryotic natural products.

Video Abstract

**Supplementary Information:**

The online version contains supplementary material available at 10.1186/s40168-023-01573-3.

## Background

Secondary metabolites are biosynthesized from primary metabolites by organisms for self-defense from environmental stress and pathogens. Marine organism-derived secondary metabolites have shown tremendous potential in drug discovery, and more than 30 compounds have been clinically approved for different kinds of medical challenges, or at different stages of clinical trials [1, 2]. Microorganisms inhabiting the marine environment not only play a critical role in the biogeochemical cycle but also produce a variety of secondary metabolites with significant biological functions [3, 4]. Marine microbes are estimated to produce more than 23,000 bioactive secondary metabolites and continue to be a treasure trove of bioactive natural products in the next century [5].

The discovery of marine prokaryotic secondary metabolites has received increasing attention in recent years and marine bacteria-derived secondary metabolites are found to show a higher chemical diversity than those of terrestrial microorganisms [6], but the number of marine prokaryotes-derived secondary metabolites is still very limited. Therefore, Voser et al. recommended several strategies for the targeted discovery of unique marine microbial secondary metabolites, including the application of cheminformatics and genome mining tools [6]. For example, Global Natural Products Social Molecular Networking (GNPS), a powerful cheminformatics platform, has been widely used for the discovery of novel molecules from diverse natural sources [7–10]. GNPS not only expedites the structural elucidation of unknown compounds but also informs other initiatives able to unveil otherwise easily overlooked compounds. As more and more comprehensive natural product structural databases (e.g., CMNPD, NPASS, and The Natural Products Atlas) are developed and released [11–14], cheminformatics strategies are expected to play an important role in the discovery of novel natural products. Besides, genome mining approaches on a global and/or large-scale basis also greatly improve the novel natural product discovery efficiency and accelerate metabolite identification in natural product research in recent decades [15–17]. The diversity of secondary metabolite biosynthetic gene clusters (BGCs) in genome-sequenced bacteria will provide crucial information for the efficient discovery of novel secondary metabolites [18]. For instance, Wang et al. identified a naturally occurring analog of colistin that could effectively eradicate the drug-resistant *Acinetobacter baumannii* by analyzing the polymyxin/colistin-like BGC in 10,858 bacterial genomes [15]. A fascinating study recently investigated the biosynthetic potential of the global ocean microbiomes and identified a di-phosphorylated product from “*Candidatus Eudoremicrobiaceae*” that belongs to an uncultivated bacterial phylum [19], suggesting the great potential of the underexplored marine microbial groups to synthesize novel bioactive secondary metabolites.

Biosynthetic chemical space refers to all possible secondary metabolites encoded by an organism [20]. Investigating the biosynthetic chemical space of marine prokaryotes will unveil the exact potential of marine prokaryotes for discovering novel secondary metabolites and figure out the microbial groups worthy of priority exploration, which will be of great reference value for the formulation of policies and guidance for the exploration of marine microbial resources.

Therefore, in this study, we aimed to quantitatively evaluate the biosynthetic chemical space of marine prokaryotes by comparing the numbers of biosynthetic gene cluster families (GCFs) and the molecular families (MFs) of reported marine prokaryotic natural products. Since the core peptides encoded by ribosomally synthesized and post-translationally modified peptide (RiPP) BGCs can be automatically obtained using bioinformatic tools and their antimicrobial potential can be reliably predicted using newly developed deep-learning algorithms [21, 22], the potential of marine prokaryotes to produce novel antimicrobial peptides from the predicted RiPPs was systematically evaluated. The diverse biosynthetic chemical space of the marine prokaryotes was experimentally exemplified by an untargeted metabolomics analysis of a sea-water-derived *Aquimarina* strain under various culture conditions.

## Methods

### Collection of marine prokaryotic genomes

A total of 42,797 marine bacterial or archaeal genomes, including 2920 culture genomes, 34,594 metagenome-assembly genomes (MAGs), and 5283 single amplified genomes (SAGs), were collected from five independent studies [3, 19, 23–25]. The quality of the genomes was estimated using CheckM v1.2.2 [26] and only genomes with completeness of ≥ 50% and contamination of < 10% were retained for downstream analyses. The detailed flowchart for genome collection can be found in Fig. S1 and the genomes used for downstream analyses were listed in Table S1**.** Finally, a total of 33,904 medium-to-high-quality marine prokaryotic genomes, including 2867 culture genomes, 26,363 MAGs, and 4674 SAGs, were included to evaluate the secondary metabolic potential of marine prokaryotes.

### Comparing the numbers of GCFs and MFs derived from marine prokaryotes

Similar to our recent study [27], all genomes were processed using the command-line version of antiSMASH v6.0.1 with the bacterial setting and otherwise default parameters [28]. The number of each class of BGCs and the necessary information regarding the composition of the BGCs in these genomes were extracted from the HTML files (antiSMASH outputs) using a customized Python kit (https://github.com/BioGavin/wlabkit). All the information on 70,011 BGCs can be found in Table S2.

To determine a suitable pair of clustering thresholds for BGCs and reported marine prokaryotic natural products, we investigated the relationship between the chemical similarity and BGC similarity for a set of 1087 bacterial BGCs with known metabolites. The structures of known compounds were converted to chemical fingerprints by a customized Python kit (https://github.com/BioGavin/wlabkit) using the Morgan fingerprint with a radius of 2 [29]. The Tanimoto coefficient of each pairwise of compounds was calculated and applied to construct the network [30]. Molecules with a Tanimoto similarity higher than the set threshold will be linked together to form an MF. The median Tanimoto similarity of known compounds within GCFs and MFs at each clustering threshold was determined and plotted in Fig. 2A, leading to the discovery of a proper MF similarity threshold that resulted in similar levels of chemical similarity represented by GCF and MF metabolite scaffolds.

Therefore, the diversity and novelty of all eight classes of BGCs, including terpene, RiPPs, nonribosomal peptide synthase (NRPS), type I polyketide synthases (PKSI), other polyketide synthases (PKSother), PKS-NRP_Hybrids, saccharides, and others, were compared with known BGCs in the MIBiG database using BIG-SCAPE at a cut-off of 0.3 [31]. Each node represents a BGC and the BGCs with similar Pfam domain units are connected by edges. The distances for each cut-off value will be used to automatically define GCFs. A molecular network with the 2884 reported marine prokaryotic metabolites, as well as 10,757 dereplicated bacterial metabolites (Table S3**)**, was constructed using the method mentioned above at a cut-off of 0.55 [12, 13]. The final similarity network was visualized using Cytoscape 3.6.1 [32].

### Investigating the diversity and novelty of GCFs

The novelty of GCFs was estimated with the total blast scores against the characterized BGCs in the MIBiG database [33] using the antiSMASH function knownclusterblast [28]. For each of the 24,536 GCFs (Table S4), the maximum total blast scores of each BGC were then averaged per GCF. An average total blast score of 0 indicates that the GCF was considered to be novel. The longest BGC in each GCF was selected as the representative BGC, and information regarding the genome type, taxon, BGC class, and the completeness (located on the edge of the contigs or not) of the representative BGC were also collected. The number of BGCs in each GCF and the average length of those BGCs were combined with the necessary information regarding the representative BGCs for GCF clustering. GCF clustering was performed using hierarchical clustering in R environment version 3.6 [34] and visualized in iTOL [35].

### The potential of marine prokaryotes to produce novel antimicrobial peptides (AMPs)

The potential of marine prokaryotes to produce novel AMPs was estimated by predicting AMPs from the putative core peptides of RiPPs BGCs using a unified deep-learning pipeline. The predicted core peptide sequences of RiPPs BGCs in all marine prokaryotic genomes were extracted from the GBK files (antiSMASH outputs) using a customized Python kit (https://github.com/BioGavin/wlabkit) and submitted to a recently developed deep-learning pipeline for candidate AMP identification, which combined multiple natural language processing neural network models, including LSTM, attention, and BERT [22]. All the predicted core peptides and AMPs of RiPPs BGCs were listed in Table S5.

### Untargeted metabolomic analysis of Aquimarina muelleri DSM 19832 ^T^

The marine sea-water-derived bacterium *A. muelleri* DSM 19832 ^T^ was obtained from the German Collection of Microorganisms and Cell Cultures GmbH (DSMZ). The complete genome sequences were re-determined using Illumina MiSeq and MinION (Oxford Nanopore Technologies) technologies. The chemical space of this strain was investigated by untargeted LC–MS-based metabolomic analysis towards different metabolic profiles of crude extracts obtained from cultivation-dependent approaches (media alteration coupled with chemical elicitors screening). The secondary metabolite production of strain DSM 19832 ^T^ under a total of 60 laboratory cultural conditions was evaluated. To be more specific, strain DSM 19832 ^T^ was cultured under 5 different liquid media (M1 ~ M5) for 3 days, then 11 chemical elicitors namely LaCl_3_·H_2_O, ScCl_3_·6H_2_O, N-acetylglucosamine, sodium butyrate, streptomycin, CoCl_2_, NiCl_2_, EDTA, DMSO, kanamycin, and ethanol were added and cultured for another 5 days, respectively. The blank and control groups were set for subsequent analysis meanwhile. Additional details of the experiment are available in Supplementary Information.

### UPLC-QTOF-MS/MS analysis

The culture broth was extracted by ethyl acetate (Supplementary Information) and then analyzed by a SCIEX X500B Q-TOF spectrometer coupled to an ExionLC AC system under the following LC conditions: column temperature of 40 ℃, flow rate of 0.3 mL/min, elution gradient: 0–2 min, 10% mobile phase A (methanol) and 90% mobile phase B (H_2_O), 2–18 min, 10–100% methanol, 18–22 min, 100% methanol, 22.01–25 min, 10% methanol. Q-TOF MS settings during the LC gradient were as follows: positive ion mode mass range 200–1500 m*/z*, total scan time 0.495 s, maximum candidate ions 5, and ion source temperature 600 ℃. MS^2^ fragmentation was achieved with Q-TOF mass range 50–1000 m*/z*, fixed collision energy 30 V, fixed collision energy spread 10 V, and ion spray voltage 5.5 kV.

The raw LC–MS data files were converted to.mzML format using MSConvert software [36] and subsequently processed using MZmine2 software [37]. Feature detection, isotope grouping, and alignment were performed following the feature-based molecular networking (FBMN) documentation [38]. The data were filtered by removing all MS/MS peaks from blank mediums. A CSV file and an MGF file were generated from MZmine2, which were uploaded and used in the FBMN workflow in GNPS (http://gnps.ucsd.edu). Molecular networks were generated with a minimum of four matching peaks, a cosine score of 0.6, and a maximum of 250 connected component sizes. In addition, the MGF file was uploaded to the Dereplicator + workflow in GNPS (http://gnps.ucsd.edu) with default parameters [39]. The molecular network from FBMN was also visualized using Cytoscape 3.6.1. Further, the metabolites were also tentatively annotated using SIRIUS 4 [40]. The mirror match of spectra was constructed using a Python package spectrum_utils [41], and the cosine value was obtained from FBMN results. All 1841 MS/MS peaks and detailed annotation results were listed in Table S7.

## Results

### The overall distribution of BGCs in marine prokaryotes

**Fig. 1:**
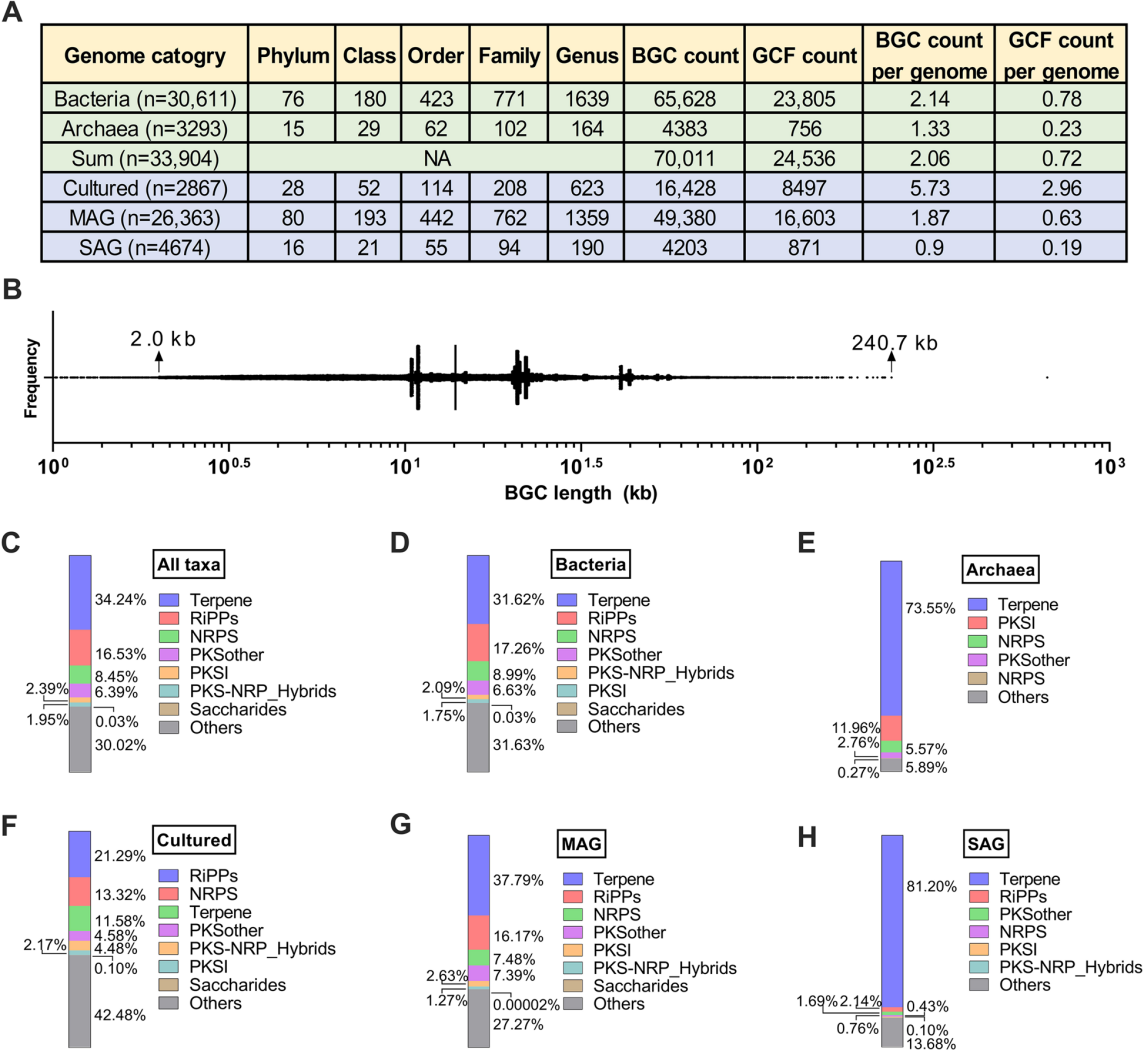
**A** Summary of unique taxa, BGCs, and GCFs across all marine prokaryotic genomes of different categories. **B** Length distribution of the BGCs in the marine prokaryotic genomes. Bar chart depicting the composition of BGCs within genomes of different categories, **C** all taxa, **D** bacteria, **E** archaea, **F** cultured genome, **G** MAG, and **H** SAG

Despite the availability of more than 220,000 bacterial genomes in the NCBI RefSeq database, only 2920 cultured genomes were derived from marine samples. Therefore, we also curated four data sets of marine prokaryotic genomes, including 34,594 MAGs from three recent large-scale metagenomics studies [19, 24, 25] and 5283 SAGs [19]. Since the assembly quality of the genomes greatly affects the predicted number of BGCs using tools like antiSMASH and our preliminary investigation suggested the completeness of 50% is a relatively appropriate threshold, which can ensure that the number of genomes is sufficient without causing too many biases in the number of BGCs (Fig. S2). Therefore, the final marine prokaryotic datasets included 2867 cultured genomes, 26,363 MAGs, and 4674 SAGs that have a completeness of ≥ 50% and contamination of < 10%. The dataset consists of 30,611 bacterial genomes and 3293 archaeal genomes (Fig. 1A and Table S1), covering a broad phylogenetic range and a large part of uncultivated microorganisms. These genomes yield an output of 70,011 BGCs mainly ranging from 2.0 to 240.7 kb in length (Fig. 1B). It is noted that the average number of BGCs in cultured genomes (5.73) is significantly larger than that in MAGs or SAGs (0.90–1.87) (Fig. 1B), implying that the secondary metabolic potential of uncultured microbiomes may be underestimated and the continuing accessing cultured genomes of marine prokaryotes remains an important object of study. The top three dominant classes of BGCs in marine prokaryotes are terpene (23,976, 34.24%), RiPPs (11,572, 16.53%), and NRPS (5914, 8.45%) (Fig. 1C), which is quite similar to the distribution of BGCs in marine bacteria (Fig. 1D).

Interestingly, 73.55% of all the predicted BGCs in archaeal genomes encode terpenes, more than six times the size of the next largest class (T1PKS) (Fig. 1E). In-depth analysis revealed that the phenomenon was mainly due to the large number of terpene BGCs in phylum *Thermoplasmatota* (Table S2), suggesting the promising potential of *Thermoplasmatota* strains to synthesize terpenoids. In addition, the composition of the seven dominant classes of BGCs in different genome types of marine prokaryotes was also compared (Fig. 1F–H), and the results demonstrate that marine prokaryote-derived SAGs also contain a large proportion of terpene BGCs, but the main contributors are *Cyanobacteriota* strains (Table S2). To eliminate the impact of the inherent redundancy of BGCs in marine prokaryotes, the 70,011 BGCs were clustered into 24,536 non-redundant GCFs at an optimized similarity threshold (0.3). The distribution of the seven dominant classes of GCFs in marine genomes of different categories is generally similar to that of the BGCs (Fig. S3). For example, the top four dominant classes of GCFs are terpene, RiPPs, NRPS, and PKSother, and the terpene GCFs are rich in *Thermoplasmatota* strains among the archaeal genomes and also predominantly observed in Cyanobacteriota strains among the SAGs. These findings further confirm the great potential for mining novel terpenoids from marine archaea.

### Biosynthetic chemical space evaluation of marine prokaryotes

**Fig. 2:**
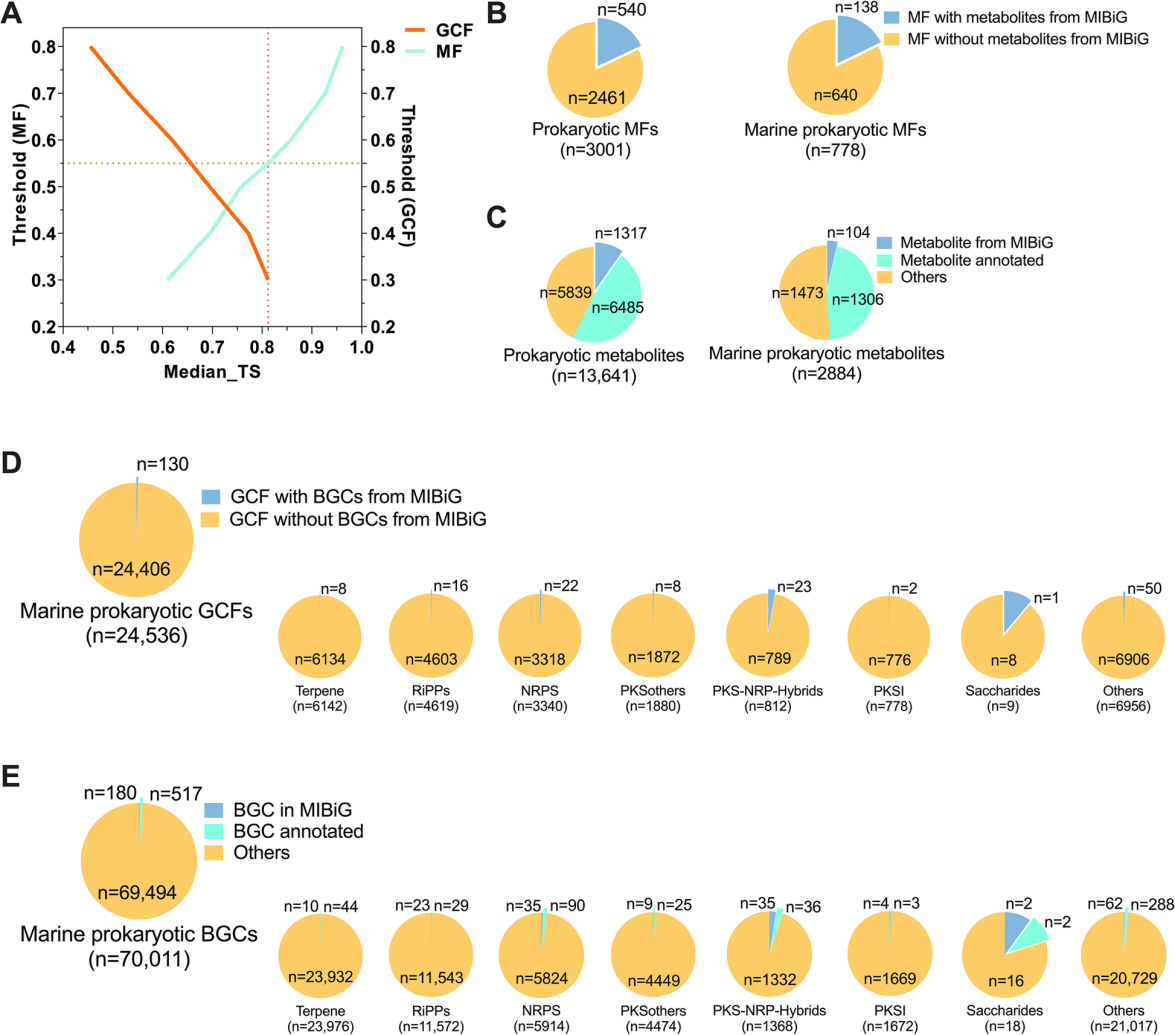
**A** The median Tanimoto similarity of known compounds within GCFs and MFs at the different clustering thresholds. A median intra-cluster Tanimoto similarity of 0.81 was chosen, corresponding to GCF and MF similarity thresholds of 0.3 and 0.55, respectively. **B** The numbers of prokaryotic and marine prokaryotic MFs with or without metabolites from MIBiG. **C** The numbers of prokaryotic and marine prokaryotic metabolites annotated or not. **D** The numbers of different classes of marine prokaryotic GCFs with or without BGCs from MIBiG. **E** The numbers of different classes of marine prokaryotic BGCs annotated or not

To quantitatively evaluate the biosynthetic chemical space of marine prokaryotes, we first determined the relationship between chemical similarity and BGC similarity for a set of 1087 bacterial BGCs with known metabolites from the MIBiG database. As shown in Fig. 2A, the median Tanimoto similarity of known metabolites within a GCF dramatically increased as the clustering threshold decreased, and an optimized MF similarity threshold (0.81) was chosen where the similarity thresholds for BGC and metabolite scaffold were 0.3 and 0.55, respectively. The thresholds enabled these elements to organize into non-redundant groups and resulted in a similar level of chemical similarity represented by GCF-encoded metabolites and MF scaffolds. The predicted BGCs were organized into 24,536 GCFs, 99.5% (24,406) of which showed a raw distance larger than 0.3 to BGCs from MIBiG (Fig. 2D and Table S4), implying that BGCs in these GCFs probably encode novel natural products. However, the reported marine prokaryotic natural products were only organized into 778 MFs at the same intra-cluster Tanimoto similarity (Fig. 2B and Table S3), suggesting that at least 96.8% (24,406 vs. 778) of the secondary metabolic potential in marine prokaryotes is untapped. Even for all the 13,641 reported prokaryotic metabolites, they were only classified into 3001 MFs (Fig. 2C), further supporting the great chemical repertoire of marine prokaryotes. To be specific, only 8 (0.11%) terpene, 16 (0.32%) RiPPs, 22 (0.63%) NRPS, 8 (0.39%) PKSothers, and 2 (0.49%) PKSI GCFs contained characterized BGCs from MIBiG. Moreover, anchoring the GCFs with BGCs from MIBiG enabled the automated annotation of 517 BGCs with predicted metabolite scaffolds (Fig. 2E), anchoring the reported prokaryotic metabolites with metabolites from MIBiG enabled the annotation of 1306 marine prokaryotic metabolites and 6485 prokaryotic metabolites with predicted BGCs (Fig. 2C). For example, several marine prokaryotic RiPP and NRP BGCs were speculated to encode Subtilosin A and Surfactin (Fig. S4). These findings would facilitate researchers to prioritize prokaryotic metabolites with unknown biosynthetic pathways and BGCs encoding promising natural metabolites.

### Diversity and novelty of marine prokaryotic BGCs

**Fig. 3:**
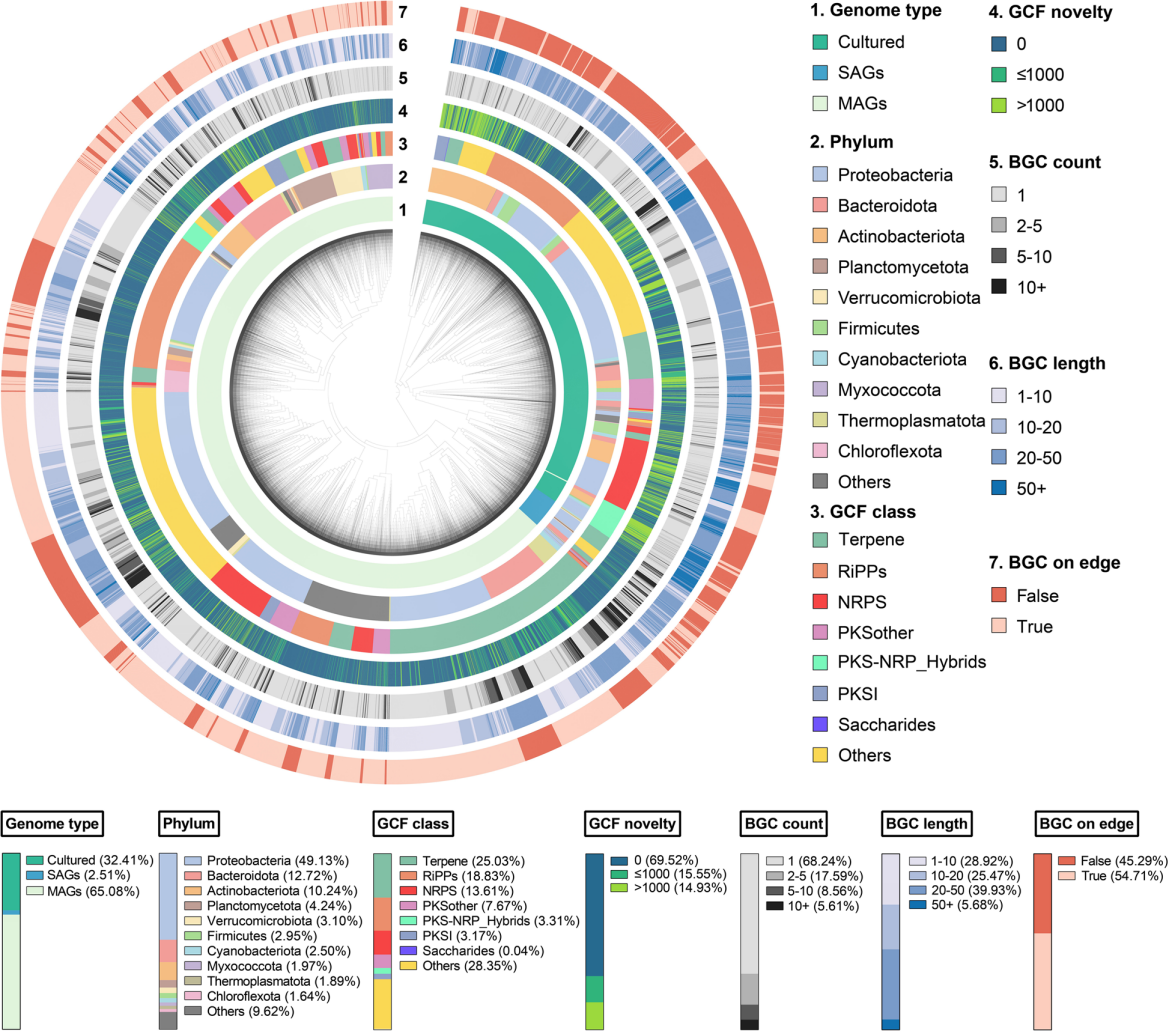
Hierarchical clustering of the 24,536 GCFs in marine prokaryotic genomes. Representation of the data (inner to outer layers): genome type and phylum level classification of organisms possessing the representative BGCs; the third and fourth layers indicate the class and novelty of GCFs; the outer three layers indicate the number of BGCs in each GCF, the average length of those BGCs, and the completeness of the representative BGCs. The proportions of each subcategory of GCFs were presented below the dendrogram

To evaluate the diversity and novelty of the marine prokaryotic BGCs, the relationships between the predicted 70,011 BGCs from marine prokaryotic genomes and known BGCs in the MIBiG database were investigated using BiG-SCAPE. The dendrogram of the resulting 24,536 GCFs was presented in Fig. 3 and Table S4, revealing that these GCFs were mainly presented in phyla *Proteobacteria*, *Bacteroidota*, *Actinobacteriota*, *Thermoplasmatota* (Archaea), *Planctomycetota*, and *Cyanobacteriota*, and with the *Proteobacteria* harboring the most diverse chemical reservoirs. Marine prokaryotes-derived MAGs captured the most diverse biosynthetic potential of marine prokaryotes, followed by the cultured genomes, which was an important complement for the global analysis of the biosynthetic chemical space of marine prokaryotes. The SAGs data mainly contributed some novel terpene GCFs from the phylum *Proteobacteria*. Moreover, RiPPs and NRPS GCFs were dominantly detected in *Proteobacteria*, while terpene GCFs were mainly observed in *Proteobacteria*, *Actinobacteriota*, *Bacteroidota*, and *Thermoplasmatota* (Archaea). Besides, 17,057 of these GCFs (69.52%) showed an average alignment score of 0 to characterized BGCs from MIBiG and only 3664 of GCFs (15.55%) showed an average alignment score larger than 1000, further implying the novelty of the marine prokaryotic BGCs. On the other hand, 68.24% of GCFs only contained one BGC and the average length of BGCs in more than 70% of GCFs was larger than 10 kb, including 5.68% of which was longer than 50 kb. These findings suggest that marine prokaryotes possess plenty of relatively complete orphan GCFs, which deserve prior exploration. Notably, more than half (54.71%) of the representative BGCs were located at the edge of the corresponding contigs and were randomly observed in different classes of GCFs, demonstrating that the fragmentation of genome sequences has implications for the mining of all classes of GCFs.

### The potential of marine prokaryotes to produce novel AMPs

**Fig. 4:**
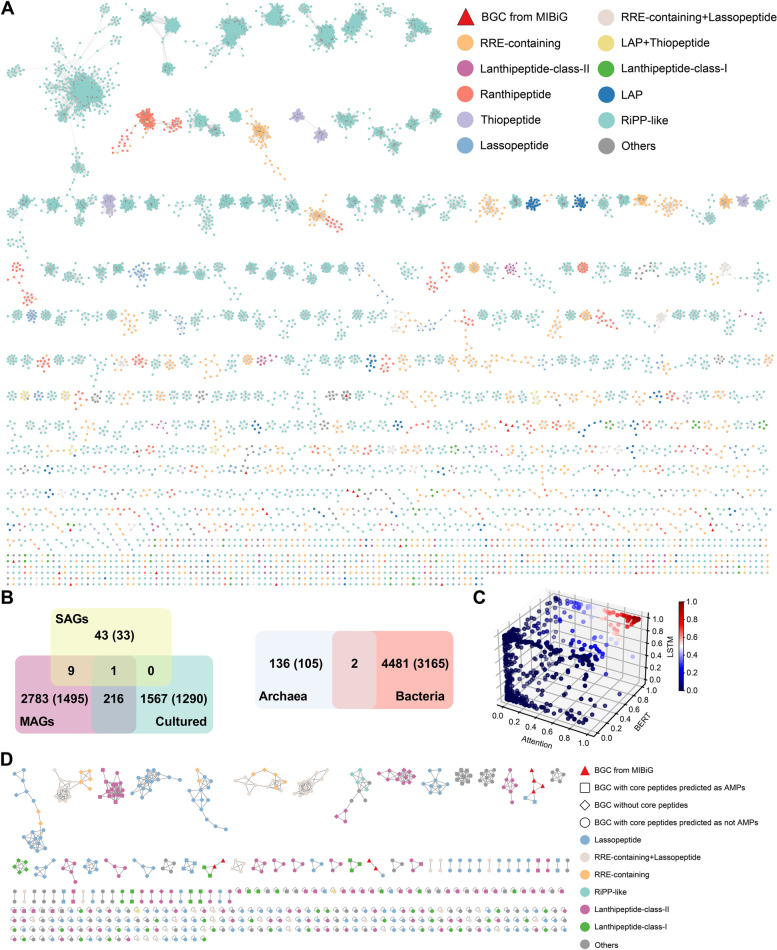
**A** Gene cluster family network of the 11,572 predicted RiPPs BGCs from 33,904 marine prokaryotic genomes, which consists of 4619 GCFs (3268 singletons not shown). The top ten dominant subcategories of RiPPs GCFs were labeled with different colors. **B** Venn diagrams of RiPPs GCF overlap in marine prokaryotic genomes of different categories. The number of GCFs and singletons (shown in parentheses) was also compared. **C** The prediction scores of the 853 core peptides in three models (attention, LSTM, and BERT).** D** GCFs containing BGCs with the core peptides can be predicted by antiSMASH. BGCs encoding core peptides predicted as AMPs were marked. Node color reflects the subcategory of RiPPs BGCs

The 11,572 predicted RiPPs BGCs from marine prokaryotes were organized into 4619 GCFs (including 3268 singletons) at the similarity threshold of 0.3 with the dominant subcategory of GCF as RiPP-like (Fig. 4A and Table S5). As shown in Fig. 4B, RiPPs GCFs were mainly distributed in MAGs (2783 unique, 60.25%) and cultured genomes (1567 unique, 33.93%), suggesting that metagenome sequencing and whole-genome sequencing are two indispensable techniques for analyzing the secondary metabolic potential of marine microorganisms. On the other hand, the majority (4481, 97.06%) of the RiPPs GCFs could be detected in bacterial genomes and only 136 (2.94%) RiPPs GCFs from archaeal genomes were unique to their counterparts. For example, BGCs in four selected GCFs were solely derived from *Thalassospira*, *Hyphomonas*, *Streptomyces*, and *Bacillus* (Fig. S5).

The core peptides of all the RiPPs BGCs were submitted to antiSMASH, leading to the discovery of 853 core peptides from 464 BGCs that are mainly derived from lanthipeptide-class-II and lassopeptide BGCs (Table S6 and Fig. 4D). The deep-learning models identified 94 core peptides as potential AMPs, including six previously reported AMPs (Subtilomycin, Aborycin, and Sviceucin, etc.) (Fig. 4C and Table S6). The other 88 core peptides showed high potential to be novel AMPs and warrant a thorough investigation. It is worth noted that the core peptides of only 4.01% of RiPPs BGCs could be predicted by antiSMASH and the potential novel AMPs were mainly identified from the cultured marine microorganism (Tables S5 & S6), implying that the cultivation of marine microorganisms is of great significance for the discovery of novel antibiotics.

### Chemical space of a sea-water-derived Aquimarina strain

**Fig. 5:**
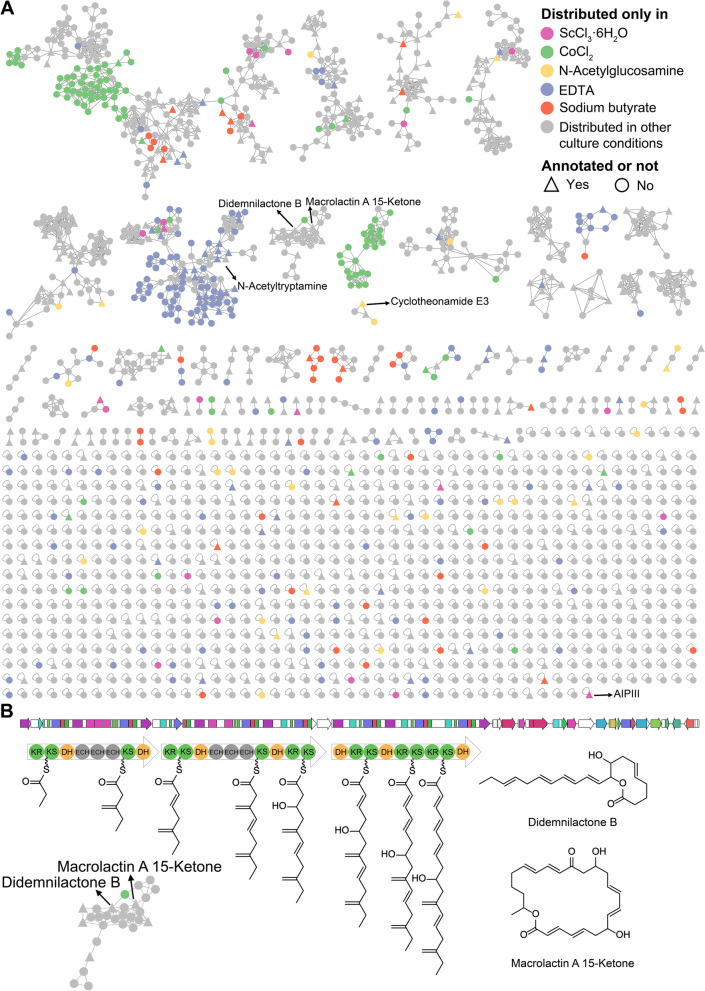
**A** Molecular network of crude extracts of *A. muelleri* DSM 19832^ T^ cultured under different culture conditions. Node color reflects whether the metabolites are produced in the presence of a specific elicitor. Node shape reflects whether the metabolites could be annotated by at least one approach. **B** The putative biosynthetic gene cluster responsible for the biosynthesis of didemnilactone B and macrolactin A 15-ketone

Among the commercially available marine-derived prokaryotes, the sea-water-derived *A. muelleri* DSM 19832^ T^ possessed many orphan and genus-specific BGCs (Fig. S6). Therefore, the chemical space of marine prokaryotes was experimentally exemplified by an untargeted metabolomics analysis of *A. muelleri* DSM 19832^ T^ under various culture conditions. The resulting mass spectra were processed with the GNPS molecular networking workflow and annotated using the FBMN workflow, Dereplicator + , and Sirius. All chemical features present in the blank samples (culture media-only) were removed during the analysis. The molecular network consisted of 1841 nodes, 56.3% of which were organized into a total of 109 MFs comprising two or more nodes each. As shown in Fig. 5A and Table S7, a total of 63 nodes could be preliminarily identified by FBMN workflow, 98 nodes were dereplicated as known metabolites using Dereplicator + (score ≥ 9), and 271 features could be relatively confidently annotated using Sirius (ConfidenceScore ≥ 0.5), demonstrating that the 1457 unannotated nodes (79.1% of the total) were potential undescribed metabolites.

Compared to blank controls, the culture broth with chemical elicitors showed upregulation of undescribed secondary metabolite production (Fig. 5A). This indicated the successful activation of cryptic BGCs by this genetic-free approach. For instance, *A. muelleri* DSM 19832^ T^ produced two known metabolites AIP-III and cyclotheonamide E3 only under the induction of ScCl_3_·6H_2_O and N-acetylglucosamine, respectively. *A. muelleri* DSM 19832^ T^ synthesized a large group of metabolites only in the presence of EDTA, which displayed high chemical similarities to an indolyl alkaloid derivative, N-acetyltryptamine (Fig. S7). The addition of CoCl_2_ induced the strain to produce at least two groups of metabolites that could not be confidently annotated by multiple approaches. In addition, *A. muelleri* DSM 19832^ T^ produced a series of compounds annotated as polyketides, including two known compounds (didemnilactone B and macrolactin A 15-ketone) (Fig. 5B and Table S7). Since the potential BGC responsible for the biosynthesis of these polyketides could not be identified from the draft genome of *A. muelleri* DSM 19832^ T^, the complete genome sequence of the strains was obtained using the MinION platform (Fig. S8). The biosynthetic gene cluster responsible for the biosynthesis of didemnilactone B and macrolactin A 15-ketone was tentatively identified according to the prediction results of the *trans*-AT PKS polyketide predictor (Fig. 5B). The discovery and biosynthetic study of the potential novel polyketide compounds deserved in-depth investigation.

## Discussion

The repeated discovery of known natural products is a major challenge in the field of natural product chemistry [42]. Marine prokaryotes exhibit great potential to synthesize novel bioactive natural products, but the detailed biosynthetic chemical space has not been comprehensively evaluated. Here, we conducted a global analysis of the diversity and novelty of the BGCs in 2867 cultured genomes, 26,363 MAGs, and 4674 SAGs of marine prokaryotes. These efforts identified 70,011 BGCs that were organized into 24,536 mostly new (99.5%) GCFs. The chemical space of marine microbiomes was quantitatively evaluated by comparing the numbers of the biosynthetic GCFs and the MFs of reported marine microbial natural products at the same intra-cluster Tanimoto similarity, revealing that more than 96.8% of the secondary metabolic potential in marine prokaryotes is untapped. Further genome mining analysis revealed the RiPPs BGCs at least encoded 88 novel potential AMPs. An untargeted secondary metabolomic study suggests the sea-water-derived *A. muelleri* DSM 19832^ T^ not only produced several known antimicrobial peptides but also had the potential to synthesize a variety of undescribed secondary metabolites.

Recently, Paoli et al. reported the biosynthetic potential of the global ocean microbiome by analyzing the diversity and novelty of BGCs in around 10,000 microbial genomes from cultivated and single cells with more than 25,000 newly reconstructed MAGs derived from the ocean [19]. At the same time, we are exploring the chemical space of marine prokaryotes using bioinformatics and cheminformatics methods. Therefore, we combined the genomic data of this study with our previous collection to evaluate the chemical space of marine prokaryotes more accurately. Finally, a total of 70,011 BGCs were obtained, which was about twice as many as the number reported in the previous study [19]. In addition, the present study focused on investigating the untapped biosynthetic chemical space of marine prokaryotes, which provides an in-depth supplement to the assessment of the secondary metabolic potential of marine microorganisms.

Although only 2867 cultured genomes (much smaller than the size of SAGs) of marine prokaryotes were included in this study, they represented a substantial proportion of the unique GCFs. While the 26,363 MAGs contributed about two-thirds of the unique GCFs, suggesting that uncultured microorganisms played a greater role in the assessment of the secondary metabolic potential of marine prokaryotes, the assembly quality of the MAGs still needs significant improvement. With the development of metagenomic sequencing and marine-specific cultivation techniques [43, 44], the following studies will continuously access complete marine prokaryotic genomes or culture genomes, thus will facilitate a more accurate evaluation of the secondary metabolic potential of marine prokaryotes.

Identifying BGCs for natural product production and predicting the structures of secondary metabolites from the BGC sequences play a crucial role in the discovery and biosynthesis of natural products [45, 46]. In this study, 517 marine prokaryotic BGCs were annotated with predicted metabolite scaffolds using the BGC clustering approach, and 6485 reported bacterial metabolites, as well as 1306 reported marine prokaryotic metabolites were connected to known BGCs or GCFs using the molecular network analysis, which provided a comprehensive perspective on the direction of marine microbial natural product discovery. Future studies may focus on the discovery of metabolites encoded by the cryptic BGCs with low similarity to known BGCs and the biosynthesis of metabolites that failed to connect to known MFs.

In the present study, we obtained 853 core peptides from the 11,572 RiPPs BGCs using antiSMASH, and 88 of them were identified as potential novel AMPs. This study strongly supported the fact that most peptides remain to be explored and the promising potential to mine novel AMPs from marine microorganisms. Our results provide much valuable information for the targeted discovery of novel AMPs and the chemical ecology studies of these peptides.

*Aquimarina* (*Bacteroidetes* phylum, *Flavobacteriaceae* family) is a marine bacterial genus that possesses a promising potential for novel secondary metabolite production, but only two compounds were isolated and characterized from *Aquimarina* strains [47–49]. *A. muelleri* DSM 19832^ T^ was isolated from a sea-water sample collected in Amursky Bay, Gulf of Peter the Great, Sea of Japan [50]. A recent metabolomics study of the extracellular *Aquimarina* extracts demonstrated that the metabolome profile of *A. muelleri* DSM 19832^ T^ scattered far from that of the other seven *Aquimarina* strains. Thirty-eight metabolites, including 6 cyclic depsipeptides, could be preliminarily identified from *A. muelleri* [51]. The present untargeted metabolomics study indicated that 79.1% of the detected metabolites of *A. muelleri* DSM 19832^ T^ under 60 different culture conditions were potentially undescribed natural products. It is worth mentioning that the metabolome profile *A. muelleri* DSM 19832^ T^ could be significantly regulated by EDTA and CoCl_2_. EDTA has been reported to induce deep-sea bacteria to produce macrolactams, daphnicyclidin alkaloids, and glycocholic acids, and CoCl_2_ can induce a marine fungus to produce polyene pyrone polyketide [52, 53]. The present study found that EDTA and CoCl_2_ induced *A. muelleri* DSM 19832^ T^ to produce a large number of undescribed metabolites, including the analogous of N-acetyltryptamine which may be synthesized through amino acid metabolism pathway, suggesting that EDTA can induce the expression of specific amino acid metabolic enzymes in marine microorganism.

## Conclusions

In conclusion, this study demonstrates that 96.8% of the secondary metabolic potential of marine prokaryotes is untapped and the unexplored biosynthetic chemical space of the marine prokaryotes was partially illustrated by the 88 potential novel AMPs from RiPPs BGCs. The untargeted secondary metabolomics study suggests a sea-water-derived *Aquimarina* strain has the potential to synthesize a variety of novel polyketide compounds. The present global bioinformatics and cheminformatics analyses elucidate marine prokaryotes’ detailed secondary metabolic potential and provide valuable knowledge for the targeted discovery and biosynthesis of marine microbial natural products.

## Supplementary Information

**Additional file 1: Figure S1.** Flowchart for the collection of marine prokaryotic genomes. **Figure S2.** (A) BGC counts, (B) ratio of BGCs on edge, and (C) number of genomes in genomes with varying degrees of completeness. **Figure S3.** Bar chart depicting the composition of GCFs within genomes of different categories, (A) all taxa, (B) bacteria, (C) archaea, (D) cultured genome, (E) MAG, and (F) SAG. **Figure S4.** A selected (A) RiPPs and (B) NRPS gene cluster family (GCF) and the comparison of the BGCs in the corresponding GCF. **Figure S5.** The predicted core peptides of BGCs in selected RiPPs GCFs. The peptide sequences from each GCF were aligned using Muscle [2] and then visualized in TBtools [3]. **Figure S6.** Gene cluster family network of the 25 predicted BGCs from *Aquimarina muelleri* DSM 19832 ^T^. *A. muelleri* DSM 19832 ^T^ possesses 20 orphan BGCs and five genus-specific BGCs (only showing a high similarity to BGCs from *Aquimarina* strains). **Figure S7.** Comparison of the experimental mass spectrum of N-acetyltryptamine with its spectrum from the GNPS database. **Figure S8.** Genomic distribution of secondary metabolite biosynthetic genes clusters in *A. muelleri* DSM 19832 ^T^. The biosynthetic gene clusters are shown in the outer layer. **Table S1.** Genomic features and the anti-SMASH results of the 33,904 marine prokaryotes-derived genomes. **Table S2.** Information about the 70,011 BGCs. **Table S3.** Information about the reported microbial natural products. **Table S4.** Information about the 24,536 GCFs. **Table S5.** Information about the RiPPs BGCs. **Table S6.** Information about the core peptides encoded by RiPPs BGCs. **Table S7.** Metabolite annotation in metabolomic data of *A. muelleri* DSM 19832 ^T^.

## Data Availability

All data generated or analyzed during this study are included in this published article and its supplementary information files.
