# Supplementary material for: Global analysis of the biosynthetic chemical space of marine prokaryotes
Source: Microbiome. 2023 Jun 28;11:144. doi: 10.1186/s40168-023-01573-3 (PMC10304266; doi:10.1186/s40168-023-01573-3)
Supplement: Supplementary file 2 — Additional file 1: Figure S1. Flowchart for the collection of marine prokaryotic genomes. Figure S2. (A) BGC counts, (B) ratio of BGCs on edge, and (C) number of genomes in genomes with varying degrees of completeness. Figure S3. Bar chart depicting the composition of GCFs within genomes of different categories, (A) all taxa, (B) bacteria, (C) archaea, (D) cultured genome, (E) MAG, and (F) SAG. Figure S4. A selected (A) RiPPs and (B) NRPS gene cluster family (GCF) and the comparison of the BGCs in the corresponding GCF. Figure S5. The predicted core peptides of BGCs in selected RiPPs GCFs. The peptide sequences from each GCF were aligned using Muscle [2] and then visualized in TBtools [3]. Figure S6. Gene cluster family network of the 25 predicted BGCs from Aquimarina muelleri DSM 19832 T. A. muelleri DSM 19832 T possesses 20 orphan BGCs and five genus-specific BGCs (only showing a high similarity to BGCs from Aquimarina strains). Figure S7. Comparison of the experimental mass spectrum of N-acetyltryptamine with its spectrum from the GNPS database. Figure S8. Genomic distribution of secondary metabolite biosynthetic genes clusters in A. muelleri DSM 19832 T. The biosynthetic gene clusters are shown in the outer layer. Table S1. Genomic features and the anti-SMASH results of the 33,904 marine prokaryotes-derived genomes. Table S2. Information about the 70,011 BGCs. Table S3. Information about the reported microbial natural products. Table S4. Information about the 24,536 GCFs. Table S5. Information about the RiPPs BGCs. Table S6. Information about the core peptides encoded by RiPPs BGCs. Table S7. Metabolite annotation in metabolomic data of A. muelleri DSM 19832 T. [file 40168_2023_1573_MOESM1_ESM.zip › 40168_2023_1573_MOESM1_ESM.pdf]

Global analysis of the biosynthetic chemical space of marine microbiomes

Bin Wei [1,2,*], Gang-Ao Hu [1,*], Zhen-Yi Zhou [1], Wen-Chao Yu [1], Ao-Qi Du [1], Cai-Ling Yang [1], Yan-Lei Yu [1], Jian-Wei Chen [1], Hua-Wei Zhang [1], Qihao Wu [4], Qi Xuan [3,#], Xue-Wei Xu [2,#], Hong Wang [1,#]

[1] College of Pharmaceutical Science & Collaborative Innovation Center of Yangtze River Delta Region Green Pharmaceuticals, Key Laboratory of Marine Fishery Resources Exploitment & Utilization of Zhejiang Province, Zhejiang University of Technology, Hangzhou 310014, China

[2] Key Laboratory of Marine Ecosystem and Biogeochemistry, Ministry of Natural Resources & Second Institute of Oceanography, Ministry of Natural Resources, Hangzhou 310012, China

[3] Institute of Cyberspace Security, College of Information Engineering, Zhejiang University of Technology, Hangzhou, 310023, China

[4] Department of Chemistry, Institute of Biomolecular Design & Discovery, Yale University, West Haven, CT 06516, United States.

[#] **Correspondence author:**

Tel: 86-571-8832-0622;

E-mail: hongw@zjut.edu.cn (H. W.); xuxw@sio.org.cn (X. X.); xuanqi@zjut.edu.cn (Q. X.)

[*] Bin Wei and Gang-Ao Hu contributed equally to this work.

METHODS

Untargeted metabolomic study of *Aquimarina muelleri* DSM 19832^T

Marine bacteria *A. muelleri* DSM 19832^T was activated and cultured in Difco Marine Broth 2216 [1] and then identified based on the 16S rDNA. For the high-throughput elicitor screening method, five different culture media were used: Difco Marine Broth 2216 (M1: peptone from soymeal 5 g, yeast extract 1 g, Fe(III) citrate 0.1 g, NaCl 19.45 g, $MgCl_2$ 5.9 g Na_2SO_4 3.24 g, $CaCl_2$ 1.8 g, KCl 0.55 g, $NaHCO_3$ 0.16 g, KBr 0.08 g, $SrCl_2$ 34 mg, H_3BO_3 22 mg, Na-silicate 2.4 mg, NaF 2.4 mg, $(NH_4)NO_3$ 1.6 mg, Na_2HPO_4 8 mg for 1 L; pH 7.6), M2 (tryptone 0.5 g, yeast extract 0.5 g, glucose 0.5 g, peptone 0.5 g, sodium 2-oxopropanoate 0.3 g, soluble starch 0.5 g, K_2HPO_4 0.3 g, $MgSO_4\cdot7H_2O$ 0.05 g, sea salt 30 g for 1 L; pH 7.5), M3 (tryptone 10 g, beef extract 3 g, sodium 2-oxopropanoate 3 g, KCl 0.2 g, sea salt 30 g for 1 L; pH 7.3), M4 (soluble starch 10 g, tryptone 0.3 g, KNO_3 2 g, $MgSO_4\cdot7H_2O$ 0.05 g, NaCl 2 g, K_2HPO_4 2 g, $CaCO_3$ 0.02 g, $FeSO_4\cdot7H_2O$ 0.01 g, sea salt 30 g for 1 L; pH 7.3), M5 (microcrystalline cellulose 10 g, peptone 0.3 g, KNO_3 0.2 g, K_2HPO_4 0.5 g, $CaCO_3$ 0.02 g, $FeSO_4\cdot7H_2O$ 0.02 g, sea salt 30 g for 1 L; pH 7.3). Pre-culture was performed in medium Difco Marine Broth 2216 for five days, after that the strain was inoculated into five different liquid mediums (M1~M5). Fermentations were performed in 12 mL cell culture tubes with 6 mL medium at 28℃ and shaking at 140 rpm for three days. Then the eleven different elicitors namely $LaCl_3\cdot H_2O$ (2 mM), $ScCl_3\cdot6H_2O$ (200 μM), N-acetylglucosamine (100 mM), sodium butyrate (100 mM), streptomycin (20

μg/mL), $CoCl_2$ (6 mM), $NiCl_2$ (100 μM), EDTA (10 mM), DMSO (3%), kanamycin (30 μg/mL), and ethanol (3%) were added and cultured for another five days. The blank groups and control groups were set for subsequent analysis meanwhile.

Sample extraction

For extraction of the liquid cultures, each 6 mL of culture broth was ultrasonicated and extracted two times with an equal volume of ethyl acetate (EtOAc), and the combined EtOAc layers were transferred into a 10 mL sample bottle and dried under vacuum. The crude extracts were re-dissolved in 0.5 mL MeOH and transferred into 1.5 mL centrifuge tubes, and concentrated with a termovap sample concentrator. The concentrated extracts were re-dissolved in 50 μL MeOH and transferred into 1.5 mL vials. The dissolved extracts were centrifuged (12,000 rpm, 10 min) and the upper layer was filtered through a 0.22 μm nylon syringe filter before injection.

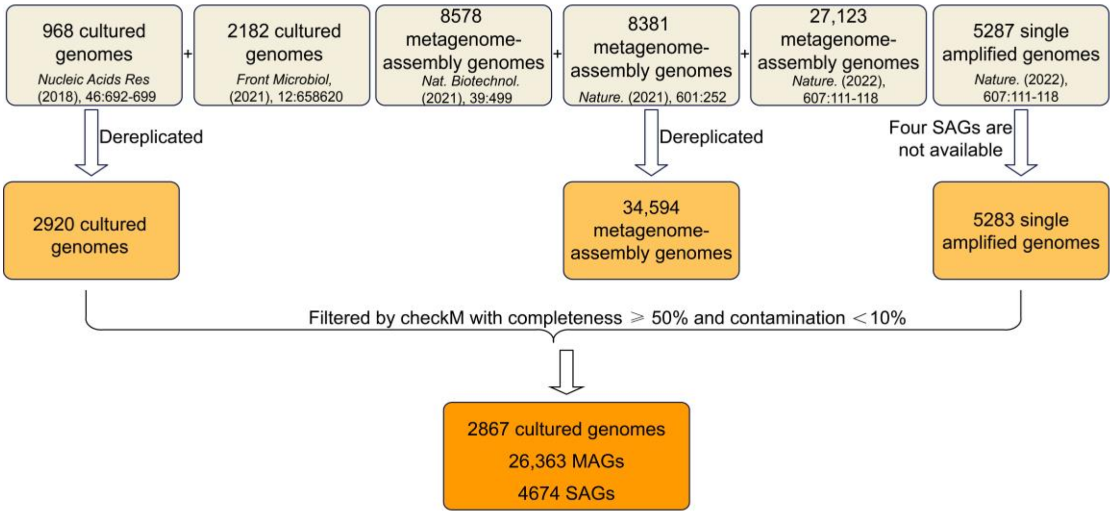

Figure S1. Flowchart for the collection of marine prokaryotic genomes

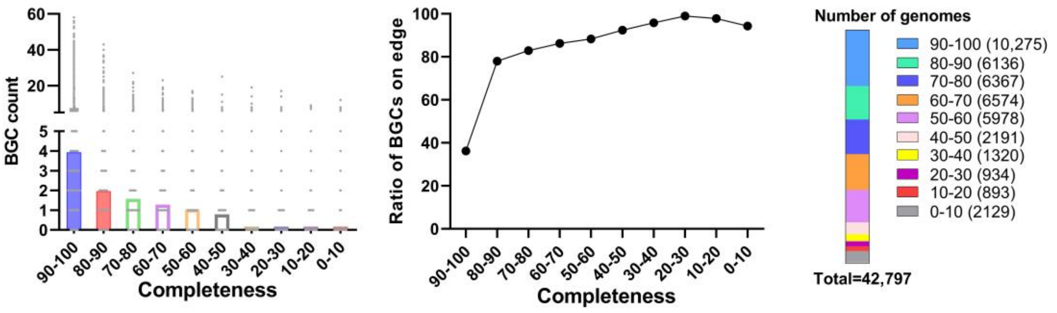

Figure S2. (A) BGC counts, (B) ratio of BGCs on edge, and (C) number of genomes in genomes with varying degrees of completeness.

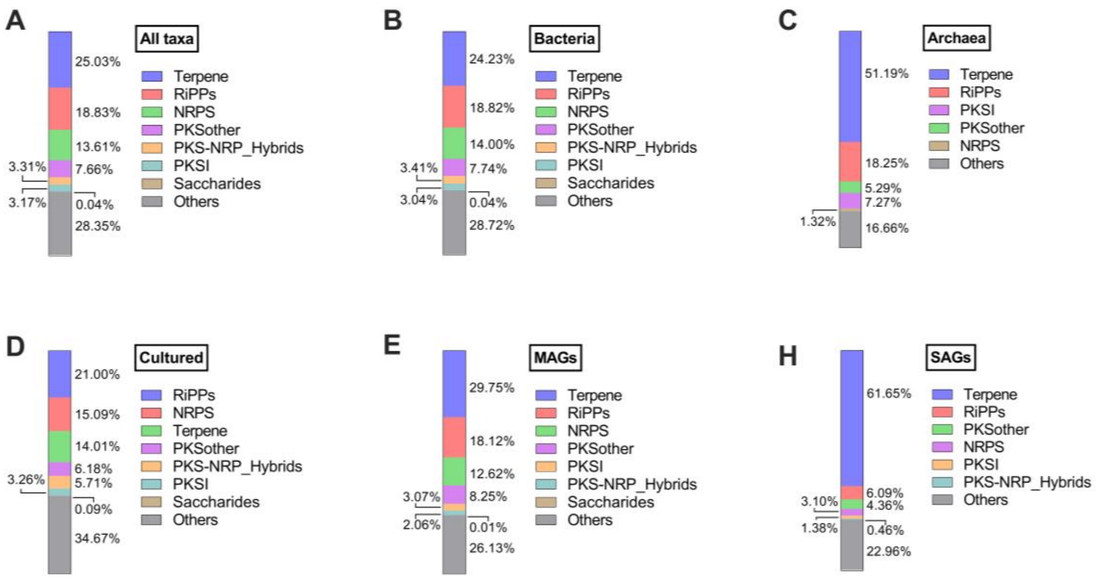

Figure S3. Bar chart depicting the composition of GCFs within genomes of different categories, **(A)** all taxa, **(B)** bacteria, **(C)** archaea, **(D)** cultured genome, **(E)** MAG, and **(F)** SAG.

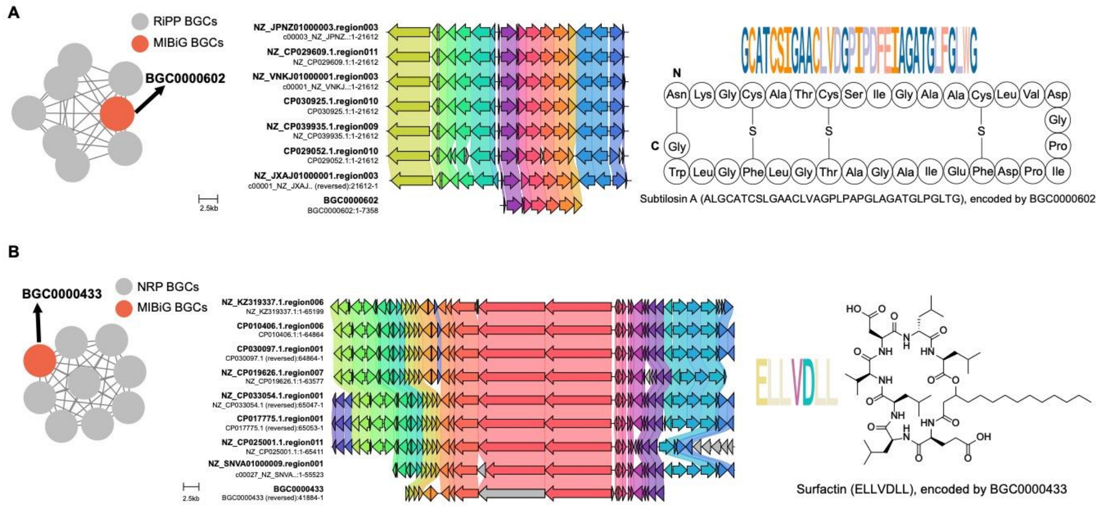

Figure S4. A selected (**A**) RiPP and (**B**) NRP gene cluster family (GCF) and the comparison of the BGCs in the corresponding GCF.

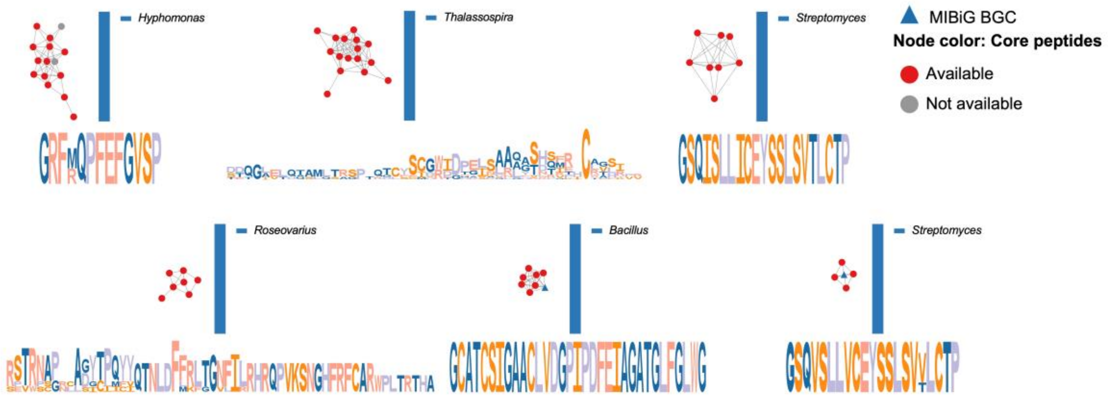

Figure S5. The predicted core peptides of BGCs in selected RiPP GCFs. The peptide sequences from each GCF were aligned using Muscle [2] and then visualized in TBtools [3].

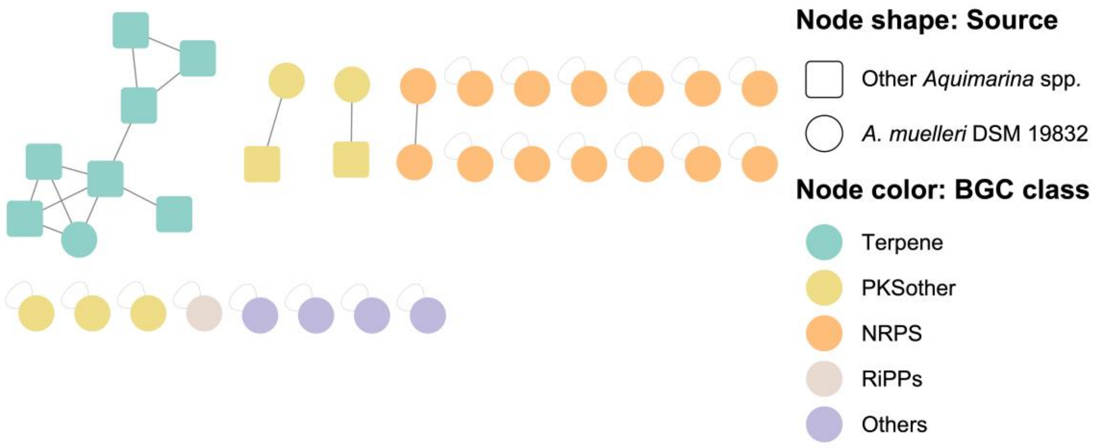

Figure S6. Gene cluster family network of the 25 predicted BGCs from *Aquimarina muelleri* DSM 19832^{T}. *A. muelleri* DSM 19832^{T} possesses 20 orphan BGCs and five genus-specific BGCs (only showing a high similarity to BGCs from *Aquimarina* strains).

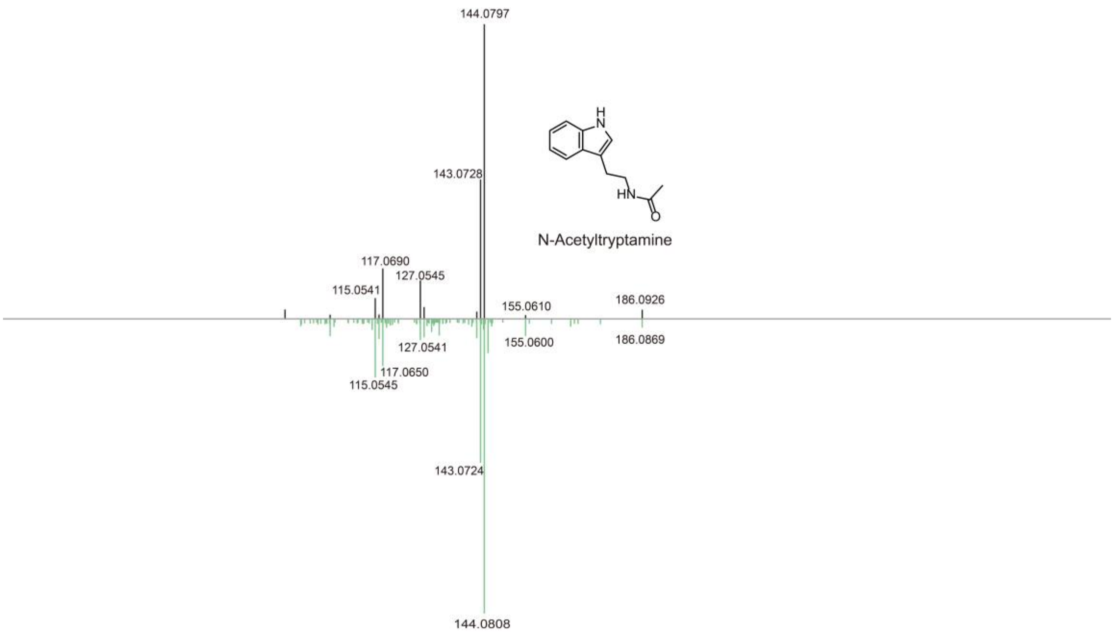

Figure S7. Comparison of the experimental mass spectrum of N-acetyltryptamine with its spectrum from the GNPS database.

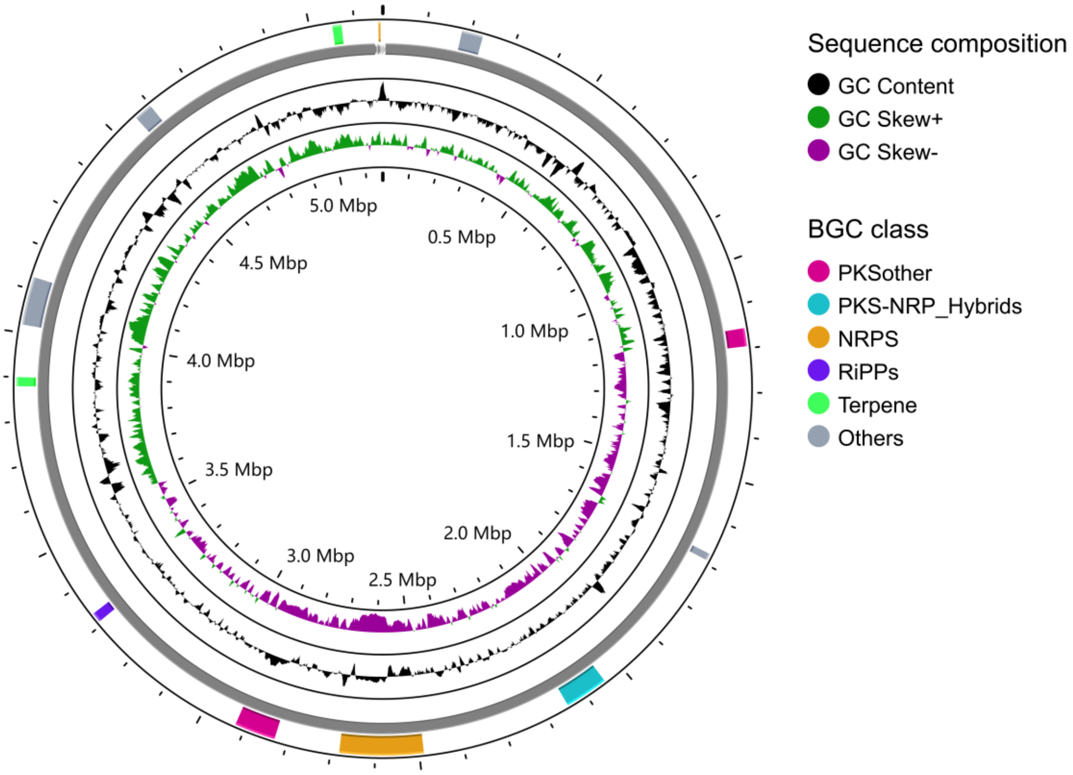

Figure S8. Genomic distribution of secondary metabolite biosynthetic genes clusters in *A. muelleri* DSM 19832[T]. The biosynthetic gene clusters are shown in the outer layer.

REFERENCES

[1] Hansen GH, Sørheim R. Improved method for phenotypical characterization of marine bacteria. J Microbiol Methods 199;13(3):231-41.

[2] Edgar RC. MUSCLE: a multiple sequence alignment method with reduced time and space complexity. BMC bioinform 2004;5(1):1-9.

[3] Chen C, Chen H, Zhang Y, Thomas HR, Frank MH, He Y, Xia R. TBtools: an integrative toolkit developed for interactive analyses of big biological data. Mol Plant 2020;13(8):1194-202.
